# Dismantling the *Beania magellanica* (Busk, 1852) species complex (Bryozoa, Cheilostomata): two new species from European waters

**DOI:** 10.1007/s12526-018-0925-2

**Published:** 2018-11-06

**Authors:** Javier Souto, Karine B. Nascimento, Oscar Reverter-Gil, Leandro M. Vieira

**Affiliations:** 1 Institut für Paläontologie, Geozentrum, Universität Wien, Vienna, Austria; 2 Facultade de Bioloxía, Departamento de Zooloxía, Xenética e Antropoloxía Fisica, Universidade de Santiago de Compostela, Santiago de Compostela, Spain; 3 Centro de Biologia Marinha, Universidade de São Paulo, São Sebastião, SP Brazil; 4 Museo de Historia Natural da Universidade de Santiago de Compostela, Parque Vista Alegre s/n, 15705 Santiago de Compostela, Spain; 5 Laboratório de Estudos de Bryozoa, Departamento de Zoologia, Centro de Biociências, Universidade Federal de Pernambuco, Recife, PE Brazil

**Keywords:** North Atlantic, Mediterranean, Chile, Spain, Cryptogenic

## Abstract

New research on bryozoans has determined that formerly widespread species are in many cases complexes of similar, but distinct, species with more restricted distributions. Notwithstanding, the limits of distribution are still unresolved for many taxa, and occasionally a wide distribution is confirmed. *Beania magellanica* has been considered a widespread species, distributed throughout the Southern Hemisphere, parts of northern Pacific and Atlantic Oceans and the Mediterranean Sea. This study examines the Magellanic-type material, together with other historic samples and new specimens collected in the western Mediterranean and Adriatic, and for the first time, presents specimens from the European North Atlantic. Morphological comparisons and biometric analysis show the existence of three different species among the specimens studied. A redescription of *B. magellanica* based on the type specimen is presented, and two new species are described: *B. serrata* sp. nov. from the Northeast Atlantic and *B. mediterranea* sp. nov. from the Mediterranean Sea. These results indicate that *B. magellanica* s.l. is a large complex of species and that most specimens from different parts of the world must be revised.

## Introduction

In the past, many bryozoan species were considered to be widely distributed or even cosmopolitan. Examples of such species are abundant in literature from the nineteenth century and early twentieth century when, for instance, many European species were cited from different localities all around the world (e.g., Busk 1884; Harmer 1926, 1957). In some cases, these were simply misidentifications; but in many cases, cosmopolitan or widespread species reflect the failure to detect subtle diagnostic differences between very closely related species (Harmelin et al. 2012). In recent years, the use of modern techniques such as SEM, the redescription of the type material and to a lesser extent in bryozoans, gene sequencing has allowed splitting of putatively widespread species into complexes of similar but distinct species with more restricted geographical ranges (Berning and Kukliński 2008; Reverter-Gil et al. 2011; Vieira et al. 2013, 2014). These new findings seem more representative for the pattern of reproduction observed in bryozoans, which are colonial animals with a brooding reproductive mode that mostly produce non-feeding larvae with a short-lived pelagic phase and consequently with a limited range of larval dispersal (MacKinney and Jackson 1989).

Nevertheless, in some species, the issue of the wide distribution remains unresolved (Harmelin et al. 2012; Cumming and Tilbrook 2014). In some species, wide geographical distributions have been confirmed, for example, in several species of the genera *Bugula* Oken, 1815 (Fehlauer-Ale et al. 2014; Ramalhosa et al. 2017), *Bugulina* Gray, 1848 (Ramalhosa et al. 2017), and *Watersipora* Neviani, 1896 (Vieira et al. 2014). Shallow-water bryozoans can be dispersed by rafting on natural floating substrates such as macroalgae, but also on artificial substrates of anthropogenic origin including drifting debris or the hulls of commercial or leisure vessels (e.g., Jackson 1986; Watts et al. 1998; Thiel and Gutow 2005; López-Gappa and Liuzzi 2018; Miranda et al. 2018). Even old records of widely distributed species apparently involve introduction by human activity centuries ago (Carlton 2009; Miranda et al. 2018).

*Beania magellanica* (Busk, 1852) is considered to be a widespread species, originally described by Busk (1852) from the Magellan Strait, Chile, where it was again recorded by Waters (1904) and Hastings (1943). Afterwards, it was recorded from the Atlantic Ocean in Cape Verde (Jullien 1888; Calvet 1907), Brazil (Hastings 1943), Argentina (Jullien 1888; Hastings 1943; López-Gappa 1989; Bastida et al. 1992; Moyano 2005; Liuzzi and López-Gappa 2011), Falkland Islands (Ortmann 1890; Hastings 1943; Moyano 2005), and Burdwood Bank (Hastings 1943). It was also recorded from the South African coasts (Jullien 1888; O’Donoghue and de Watteville 1944; O’Donoghue and Day 1957; Hayward 1980; Hayward and Cook 1983; Florence et al. 2007), the subantarctic region at Kerguelen (Busk 1879; Kluge 1914), Marion and Prince Edward Islands (Hastings 1943; Hayward 1995), the Pacific coast of Japan (Jullien 1888; Ortmann 1890; Yanagi and Okada 1918; Harmer 1926; Silén 1941), Mexico (Soule 1959), Peru (Osburn 1950, 1953) and Chile (Moyano 1999, 2009). In the Indo-Pacific region, *B. magellanica* was recorded from Australia (Hincks 1885; MacGillivray 1887; Harmer 1926; Vail and Wass 1981; Bock 1982; Winston 1986; Moran and Grant 1989, 1991, 1993; Connell 2001; Glasby et al. 2007; Piola and Johnston 2008), New Zealand (Busk 1852; Hutton 1891; Hamilton 1898; Waters 1906; Livingstone 1927; Gordon 1970, 1984, 1986; Bradstock and Gordon 1983; Rowden et al. 2004; Gordon et al. 2009), and New Caledonia (Gordon 2007). Finally, in the Mediterranean Sea, it has been extensively recorded from the western basin (Waters 1879, 1897; Jullien 1888; Gautier 1962; Prenant and Bobin 1966; Silén 1972; d’Hondt 1979; Zabala 1986; Saguar and Boronat 1987; Templado et al. 2002; Rosso et al. 2010), along with some records from the eastern basin, in Lebanon (Harmelin et al. 2016), and in Greece (Harmelin 1969; Hayward 1974; Gerovasileiou and Rosso, 2016), as well as in the Adriatic Sea (Heller 1867; Novosel and Požar-Domac 2001; Hayward and McKinney 2002; Novosel et al. 2004; Cocito et al. 2006; Cupido et al. 2007; Rosso et al. 2010) and African coast, mainly from Algeria and Tunisia (Gautier 1962; d’Hondt and Mascarell 2004; d’Hondt and Ben Ismail, 2008; Ayari-Kliti et al. 2012).

Here, we compare newly collected samples from off Atlantic northern Spain, the western Mediterranean Sea, and the northern Adriatic Sea with the type specimen of *B. magellanica*, material of historical collections and data from the literature. Two new species of *Beania* are described, establishing that *B. magellanica* actually represents a species complex.

## Material and methods

Samples from two localities in the north Iberian Peninsula were collected. Surveys by scuba were done at Point Etxandarri, northern Spain, during summer 2016 and at the Ría of Ferrol, NW of Spain, during spring 2017. At Point Etxandarri, bryozoan colonies were scraped from vertical walls in microhabitats formed by the erect bryozoan *Smittina cervicornis* (Pallas, 1766). Parts of the samples were fixed in 96% alcohol and other samples were dried. At the Ría of Ferrol, algae were collected and the rocks scraped. Collected material was transported in seawater to the lab at the Marine Biological Station of the University of Santiago de Compostela on Ferrol, where samples were sorted under a binocular microscope and fixed in 70% alcohol.

In the Adriatic Sea, samples were also collected by scuba from two localities along the coast of the Istria Peninsula in early June 2017. We also studied samples from the Balearic Islands and the Mediterranean Iberian coast, collected during the sampling surveys *Fauna Ibérica III* and *Fauna Ibérica IV*, and stored in the Museo Nacional de Ciencias Naturales (MNCN), Madrid. One small sample collected in Banyuls-sur-Mer (Mediterranean coast, Southern France), assembled in a preparation for microscopy and stored in the Muséum National d’Histoire Naturelle (MNHN), Paris, was also studied. Finally, we also examined the type specimen of *B. magellanica*, other specimens collected from the Magellan Strait, Argentina, Falkland Islands, and Burdwood Bank, and specimens from Brazil and the Mediterranean Sea currently stored at the National History Museum (NHMUK), London.

Detailed data about the date of the sample collection, substrate, etc., when is known, are presented in the section of material examined for each species. Specimens were examined in the lab using a Leica MZ12 stereomicroscope, and optical photos were taken with a Zeiss SteREO Discovery V20 stereomicroscope. Selected specimens were dried for study by scanning electron microscopy (SEM). FEI Inspect S50 SEM and Zeiss EVO LS15, from the University of Vienna and from the University of Santiago de Compostela, respectively, were used to take photographs of uncoated material with back-scattered electron detector in low-vacuum mode. Specimens deposited at the NHMUK were photographed under a LEICA CRT5000 stereomicroscope and SEM images were made in a LEO 1455VP microscope.

Colonies and zooids were measured using the software ImageJ® on optical and SEM photographs. A total of 211 zooids were measured: 51 from the Balearic Islands, four from Banyuls-sur-Mer, 85 from northern Spain, 51 from the Adriatic, and 20 from Chile (ten on the type specimen, NHMUK 1854.11.15.100, and ten from the sample NHMUK 1958.4.14.6). It was not possible to include measurements of all examined specimens deposited at museum collections due different states of conservation and preservation of the colonies. Measurements of the total length and width of the autozooids, length and width of tube connections, and length and width of avicularia were taken. Total length versus width of each autozooid was represented graphically to compare the distribution of measurements in relation with its locality. Non-parametric multidimensional scaling (nMDS) was performed to test the null hypothesis that there are no significant morphometric differences between the zooids from the different geographic areas. Measurements of autozooid total length and width and of avicularia length and width were used in this analysis. Additional information about the morphology and distribution of *B. magellanica* was gathered from the literature.

New samples collected at Point Etxandarri, Ría of Ferrol, and the Adriatic Sea were sent to the Museo de Historia Natural, University of Santiago de Compostela (MHNUSC), Spain. Comparative specimens from Port-Cros Island (France), donated by Jean-Georges Harmelin and recorded in Harmelin (2017), were deposited in the Museu de Zoologia, University of São Paulo (MZUSP), Brazil.

## Results

### Morphological and biometric analysis

**Fig. 1:**
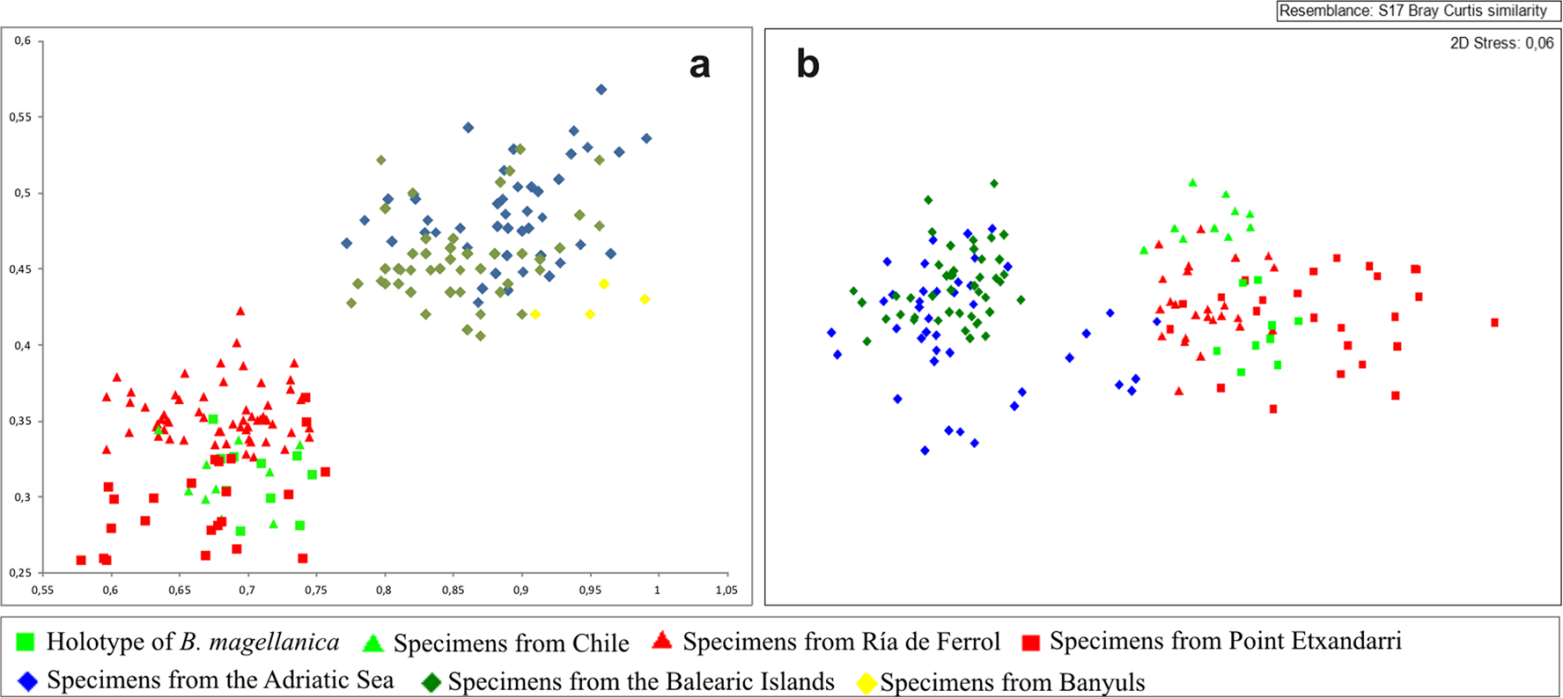
Biometric analysis of zooids by localities. **a** Graphical representation of zooidal length vs. zooidal width. **b** nMDS analysis of zooidal biometrics

Analysis of the biometric and morphological data showed clear differences between the specimens from the Atlantic coast of northern Spain, the western Mediterranean and the Adriatic, and the type material of *B. magellanica*. Measurements of the samples from the different localities showed some differentiation between autozooid size (length and width) and avicularium size (length and width) (Table 1). The most striking feature was the separation of the samples into two clear groups based on autozooid size using the biometric analysis (nMDS and long vs. wide representation) (Fig. 1). A cluster with bigger zooids is formed by specimens from the Mediterranean studied here, whereas Atlantic specimens from northern Spain and the type material did not differ significantly, forming another cluster. Furthermore, in the western Mediterranean and Adriatic colonies, true spines were not observed and only two or four shallow folds in the oral area may be present. In contrast, in the other cluster, the autozooids exhibited four short oral spines: two distal and two distolateral ones. This morphological character, together with the significant difference in zooid size, justifies the distinction of the Mediterranean material as belonging to a new, different species.

**Table 1:** Biometrics of specimens of *Beania magellanica* Busk, 1852, *Beania serrata* sp. nov. and *Beania mediterranea* sp. nov. from different localities

		Mean	SD	Minimum	Maximum	*N*
*Beania magellanica*	Holotype					
	Autozooid length	0.707	0.0263	0.675	0.747	10
	Autozooid width	0.313	0.0227	0.277	0.351	10
	Tubular connection length	0.101	0.0139	0.074	0.131	18
	Tubular connection width	0.054	0.0076	0.045	0.075	18
	Avicularium length	0.251	0.0235	0.225	0.288	9
	Avicularium width	0.097	0.0112	0.071	0.109	9
	NHMUK1958.4.14.6 Chile					
	Autozooid length	0.686	0.0313	0.635	0.738	10
	Autozooid width	0.313	0.0215	0.282	0.344	10
	Avicularium length	0.326	0.0138	0.303	0.355	10
	Avicularium width	0.115	0.0117	0.099	0.131	10
*Beania serrata n.* sp.	NW Spain, Ferrol					
	Autozooid length	0.679	0.0401	0.597	0.745	60
	Autozooid width	0.355	0.0187	0.326	0.422	60
	Tubular connection length	0.165	0.0244	0.114	0.228	38
	Tubular connection width	0.068	0.0104	0.051	0.097	38
	Avicularium length	0.276	0.0223	0.232	0.337	26
	Avicularium width	0.106	0.0103	0.085	0.132	26
	N Spain, Point Etxandarri					
	Autozooid length	0.666	0.0524	0.578	0.757	25
	Autozooid width	0.291	0.0322	0.236	0.365	25
	Tubular connection length	0.101	0.0232	0.064	0.144	19
	Tubular connection width	0.041	0.0078	0.030	0.056	19
	Avicularium length	0.243	0.0302	0.195	0.291	25
	Avicularium width	0.086	0.0131	0.061	0.106	25
*Beania mediterranea n.* sp.	Balearic Island					
	Autozooid length	0.853	0.0439	0.775	0.957	51
	Autozooid width	0.456	0.0282	0.406	0.529	51
	Tubular connection length	0.231	0.0402	0.180	0.340	22
	Tubular connection width	0.071	0.0121	0.050	0.100	22
	Avicularium length	0.367	0.0269	0.320	0.418	44
	Avicularium width	0.136	0.0131	0.117	0.173	44
	Banyuls					
	Autozooid length	0.953	0.0330	0.910	0.990	4
	Autozooid width	0.428	0.0096	0.420	0.440	4
	Adriatic Sea					
	Autozooid length	0.877	0.0556	0.742	0.991	51
	Autozooid width	0.470	0.0510	0.342	0.568	51
	Tubular connection length	0.222	0.0350	0.139	0.295	38
	Tubular connection width	0.082	0.0180	0.059	0.139	38
	Avicularium length	0.336	0.0474	0.250	0.402	41
	Avicularium width	0.121	0.0198	0.078	0.168	41

*SD*, standard deviation; *N*, number of measurements

**Fig. 2:**
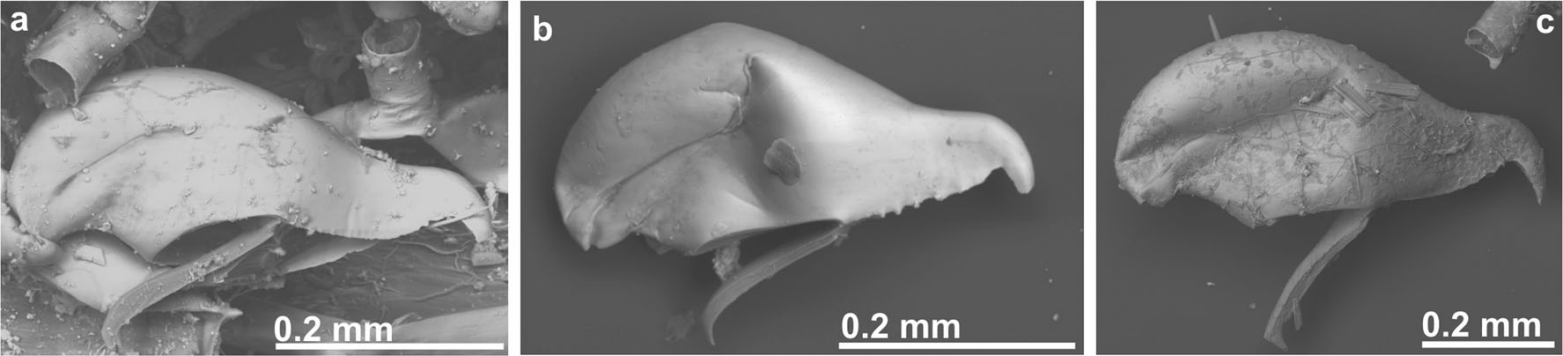
Comparison of avicularia. **a** Avicularium of *B. magellanica* Busk, 1852 (NHMUK 1996.12.11.2, Chile). **b** Avicularium of *B. serrata* sp. nov. (MHNUSC 10087, Ría de Ferrol, Spain). **c** Avicularium of *B. mediterranea* sp. nov. (MHNUSC 10104, Croatia)

In the second cluster, material from the Atlantic coast of northern Spain differed from the Magellan Strait material mainly in the morphology of the avicularium: the avicularium exhibits a serrated or denticulate distal rostrum in northern Atlantic Spain but a straight rostrum in the Magellan Strait (as is also the case in the material from the Mediterranean) (Fig. 2). Moreover, in Spanish specimens, the two distal spines are reduced or lacking in zooids with an ooecium. These differences in the Spanish Atlantic material justify the description of a new, distinct species.

### Systematics

Class Gymnolaemata Allman, 1856

Order Cheilostomata Busk, 1852

Family Beaniidae Canu and Bassler, 1927

Genus *Beania* Johnston, 1840

**Fig. 3:**
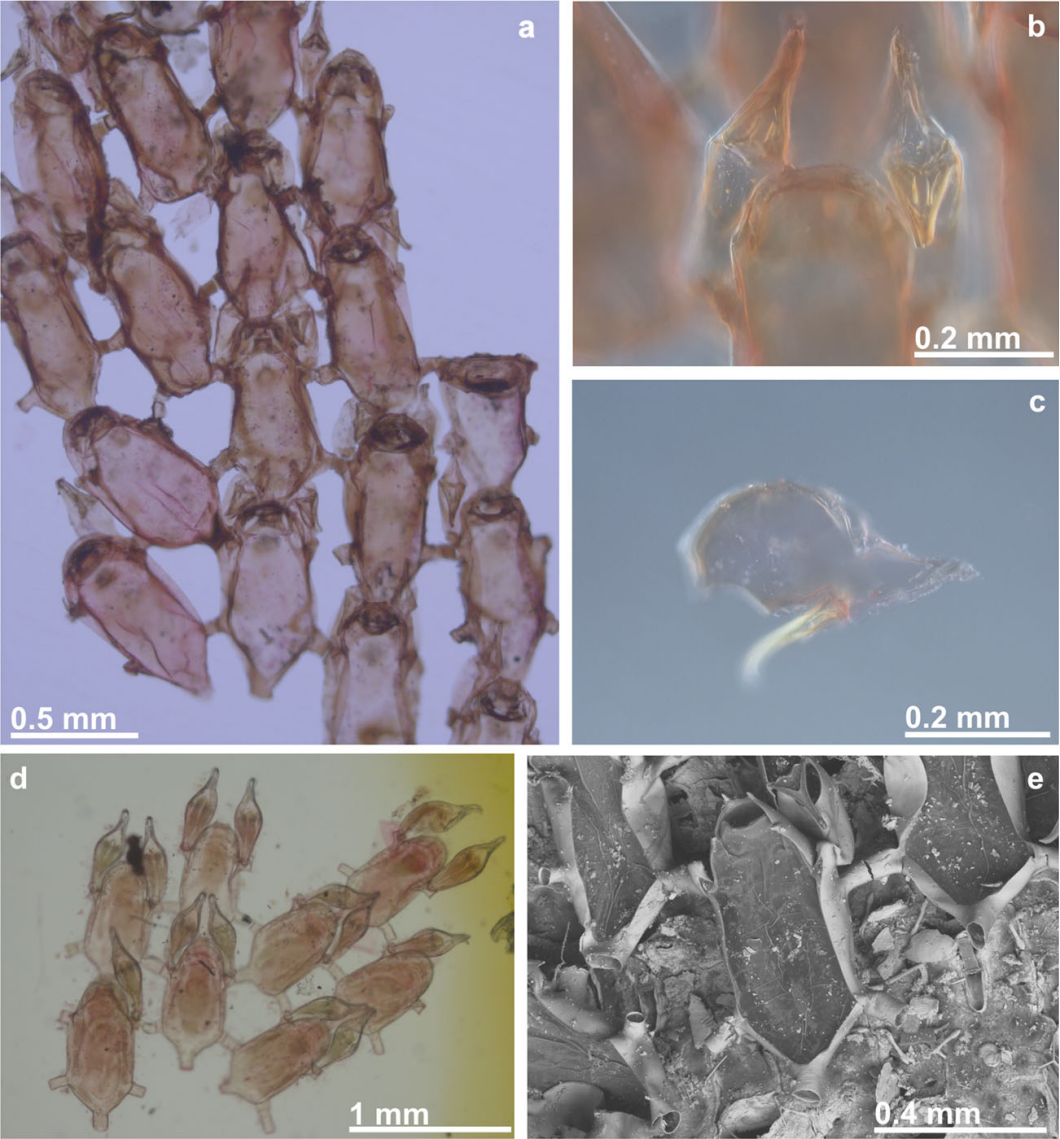
*Beania magellanica* Busk, 1852. **a**–**c** Holotype specimen from the Magellan Strait (NHMUK 1854.11.15.100): **a** general aspect of colony, **b** detail of distal portion of one zooid with two avicularia, and **c** avicularium. **d** Portion of one colony with avicularia in all zooids (NHMUK 1958.4.14.6, Chile). **e** SEM image of zooid (NHMUK 1996.12.11.2, Chile)

***Beania magellanica*** (**Busk**, **1852**)

(Figs. 2a and 3; Table 1)

*Diachoris magellanica* Busk, 1852: 54, pl. 67; Busk 1884: 59

*Beania magellanica*: Waters 1904: 28, pl. 08, fig. 7 a–c; Hastings 1943: 414

### Material examined

*Holotype*: *Diachoris magellanica*, NHMUK 1854.11.15.100, Magellan Strait, Chile, G. Busk Collection, collector Charles Darwin, 10–20-m depth.

*Other material*: **Brazil**—NHMUK 1899.7.1.4674, John Adams Bank, off Espírito Santo, G. Busk Collection. **Argentina**—NHMUK 1934.11.12.23, 51° 35′ S, 65° 39′ W, St. 314, G. Busk Collection, H.M.S. Challenger, 70-m depth; NHMUK 1967.1.2.11, 49° 47′ 45″ S, 61° 05′ W, St. WS784, Discovery Investigations, 164–170-m depth, 05/xii/1931; NHMUK 1967.1.2.11, 53° 11′ 45″ S, 65° W, St. WS838, Discovery Investigations, 148–159-m depth, 05/ii/1932; NHMUK 1996.12.4.24/25/26, 46° S, 60° 05′ W, St. WS237, Discovery Investigations, 150–246-m depth, 07/vii/1928; NHMUK 1996.12.22.4, 50° 35′ S, 57° 20′ W, St. WS229, Discovery Investigations, 210–271-m depth, 01/vii/1928. **Chile**—NHMUK 1958.4.14.6, 1996.12.11.2, Magellan Strait, St. 1321, Discovery Investigations, 66-m depth, 16/iii/1934. **Falkland Islands**—NHMUK 1887.12.9.298/300, 1944.1.8.154, Port Jackson, 51° 40′ S, 57° 50′ W, St. 315, G. Busk Collection, H. M. S. Challenger, 5–12-m depth; NHMUK 1947.4.22.207, Port Stanley, St. 58, Discovery Investigations, 1–2-m depth, 19/v/26; NHMUK 1996.12.4.14/15/18/20, off Lion Island, St. WS84, Discovery Investigations, 74–75-m depth, 24/iii/1927; NHMUK 1996.12.4.16/17/21/22/23, off Lively Island, St. WS85, Discovery Investigations, 79-m depth, 25/iii/1927; NHMUK 1930.1.16.13, Roy Cove; NHMUK 1935.3.6.327/401, Whaler Bay, Vallentin Collection; NHMUK 1935.3.6.328, Stanley Harbour, Vallentin Collection. **Burdwood Bank**—NHMUK 1996.10.11.1, St. 1909, Discovery Investigations, 132-m depth, 30/iv/1936.

### Description

Colony creeping, reticulated, forming a sheet with network aspect and supported above the substrate, and fixed to it, by smooth tubular rootlets resembling holdfast tips. Autozooids disjunct, elongate oval, boat-shaped, linked by tubular elongations of the lateral and abfrontal wall, each zooid with six tubes, one distal (in the opposite region of the opening of the operculum), two distolateral, two proximolateral, and one in the most proximal portion of the zooid, each connection with a septum. Frontal wall completely membranous, without frontal or marginal spines. Four (rarely two or none) small oral spines, two on distal corners facing up, and two distolateral. Distal end of the autozooid lightly rounded or straight, with the edge of the operculum fitting to the distal rim. One or two monomorphic avicularia, pedunculate, with bird’s head form, about half of zooidal length, attached by a short tube near each distolateral tubular connection, elongate cystid, almost funnel-shaped, with dorsal wall rounded in profile, occupying about three fifths of avicularium length, rostrum (not visible in type material) downcurved distally, smooth, tapering distally, mandible with single, hooked tip. Ooecium vestigial (not visible in type material), resembling a small cap at the distal zooidal edge, zooid with ooecium with more spaced distal spines.

### Remarks

Busk’s type is a small colony preserved on a balsam slide, making it difficult to characterize certain morphological features of this species, including avicularia and ooecia. Therefore, the above description includes data from the type specimen as well as additional material from the Magellan Strait.

**Fig. 4:**
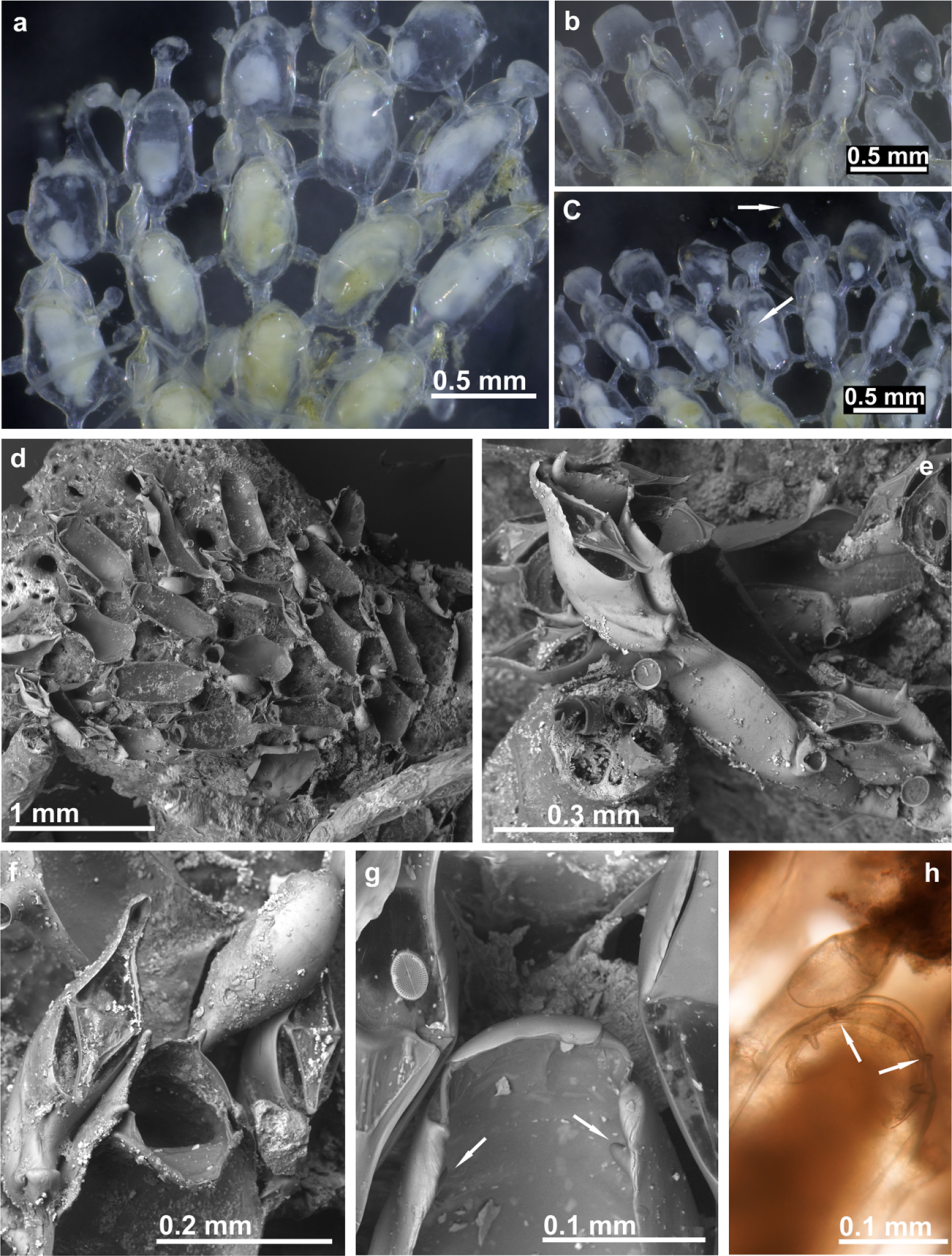
*Beania serrata* sp. nov. from Ría de Ferrol, Spain (NE Atlantic). **a**, **b** Optical frontal view of colony (MHNUSC 10087, Holotype). **c** Optical photo of basal wall, arrows show rhizoids (MHNUSC 10087, Holotype). **d** SEM image of colony (MHNUSC 10088, Paratype). **e**, **f** Zooids with distal avicularia and distal spines (MHNUSC 10088, Paratype). **g** Zooid with ooecium, arrows show proximal-most spines (MHNUSC 10088, Paratype). **h** Optical photo of distal part of one zooid with ooecium, arrows show reduced distal spines

**Fig. 5:**
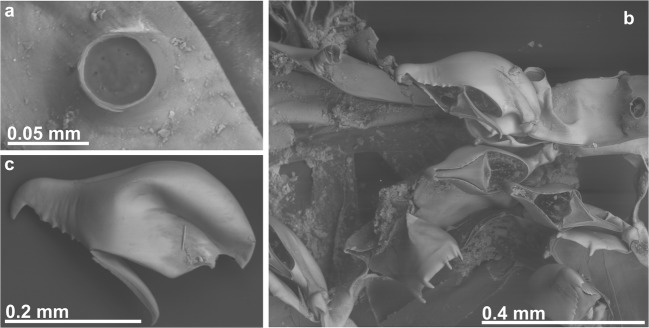
*Beania serrata* sp. nov. (MHNUSC 10087, Holotype, Ría de Ferrol, Spain). **a** Septum in a tubular connection between zooids. **b** SEM image showing avicularia and distal spines. **c** Avicularium aspect

***Beania serrata*sp**. **nov**.

(Figs. 2b, 4, and 5; Table 1)

urn:lsid:zoobank.org:act:D17A2B14-77AF-4349-B9F3-321D37D7F39E

### Material examined

*Holotype*: MHNUSC 10087, Pta. Piteira, Ría de Ferrol, Spain, NE Atlantic, 43° 27′ 52″ N, 08° 15′ 43″ W, 5–16-m depth, 25/05/2017 on *Gloiocladia repens* (C. Agardh) Sánchez and Rodríguez-Prieto in Rodríguez-Prieto et al. (2007).

*Paratypes*: MHNUSC 10088, 10089, 10090, same locality of the Holotype, MHNUSC 10106, Point Etxandarri, Spain, NE Atlantic 43° 26′ 32.1″ N, 2° 55′ 27.5″ W, 25-m depth, 04/08/2016, on *S. cervicornis* (Pallas, 1766).

*Etymology*: *serrata*, alluding to the serrate aspect of the avicularian rostrum, one of the main differences between this and the other two species described herein.

### Description

Colonies forming a sheet with network aspect, supported above the substrate, and fixed to it, by rootlets of different size, from short to more than 1 mm long, rootlets growing from the distal basal part of the zooid, below the distal tubular intrazooidal connection, with terminal end resembling a holdfast tip. Autozooids disjunct, elongate oval, boat-shaped, linked by tubular connections, each autozooid with six tubes, one distal, two distolateral, two proximolateral, and one proximal. A septum, visible by transparency, in the middle of the tubular connections, with numerous small rounded pores forming a circle. Frontal wall completely membranous. Four small spines present, two distal and two lateral to the orifice, with the two distal ones very reduced or absent in zooids with ooecium. Distal end of autozooid lightly rounded or straight, with the edge of the operculum fitting to the distal rim. One or two monomorphic avicularia, pedunculate, with bird’s head form and very mobile, about half zooidal length, attached by a short tube near each distolateral tubular connection and directed frontally, elongate cystid, almost funnel-shaped, with dorsal wall rounded in profile, rostrum downcurved distally, showing a denticulate border, with serrated aspect. Ooecium very reduced, like a small cap in the distal margin with two lateral uncalcified lines.

### Remarks

*Beania serrata* sp. nov. differs from *B. magellanica* mainly in the rostrum of avicularia having a denticulate border. Specimens of *B. serrata* sp. nov. were collected in the Atlantic, north and north-west of Spain. The bryozoan fauna of these areas was well studied in the past, but there are no previous records of *B. magellanica* here. Moreover, both areas are under high anthropogenic pressure, with large harbor areas and dense maritime traffic. Therefore, this species is considered cryptogenic until more studies can be conducted on it and related species.

**Fig. 6:**
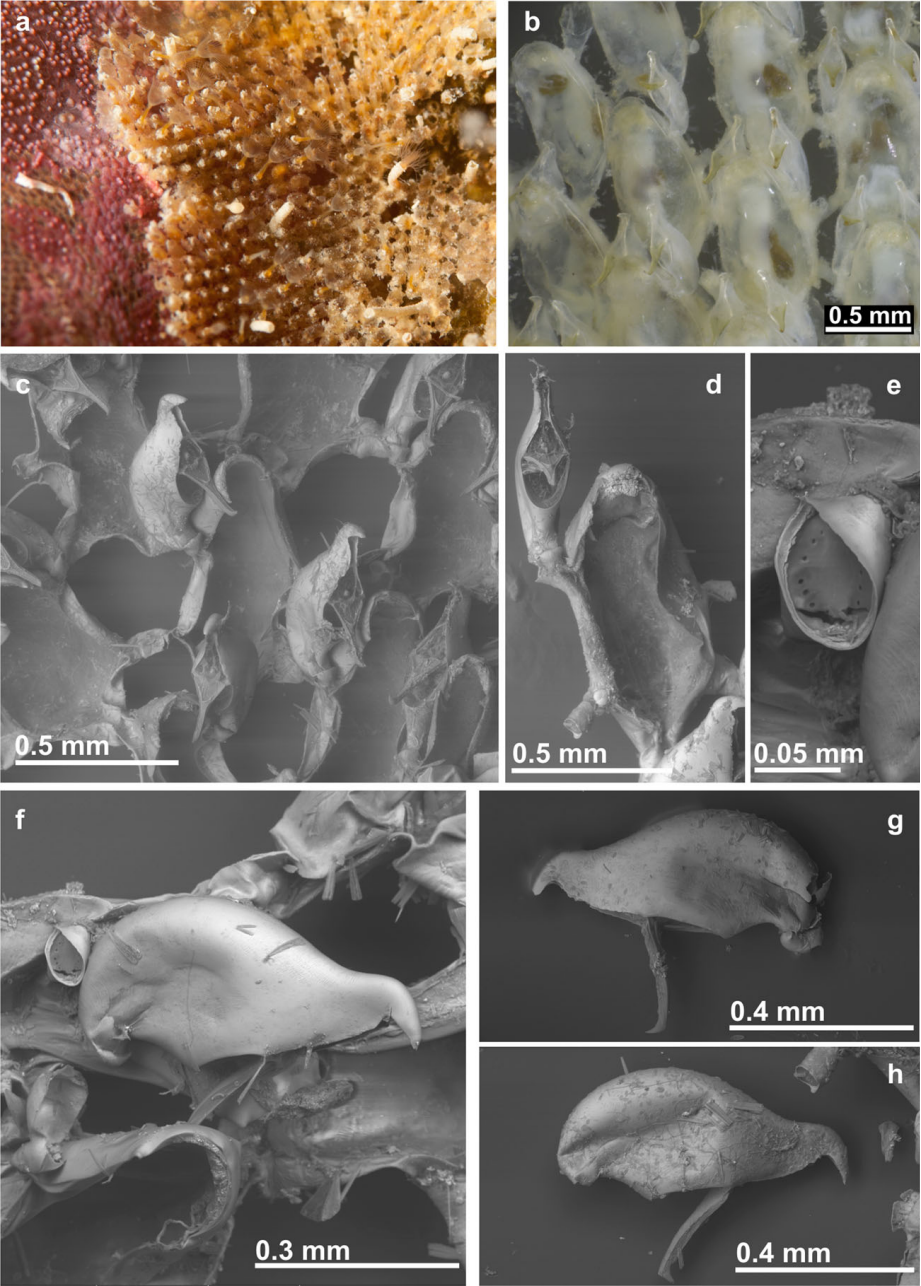
*Beania mediterranea* sp. nov. **a** Aspect of colony with tentacle crown expanded. **b** Optical photos of zooids with avicularia (MHNUSC 10105, Paratype, Croatia). **c**, **d** Zooids and avicularia in an SEM image (MHNUSC 10105, Paratype, Croatia). **e** Septum in a tubular connection between zooids (MHNUSC 10104, Holotype, Croatia). **f**–**h** Avicularia (MHNUSC 10104, Holotype, Croatia)

***Beania mediterranea*sp**. **nov**.

(Figs. 2c and 6; Table 1)

urn:lsid:zoobank.org:act:8D4CCBF4-E4BC-4EFF-A94D-A48723C74444

*Diachoris buskiana* Hutton, 1873: Waters 1879: 120, pl. 12, fig. 1

*Beania magellanica*: Waters 1897: 16, pl. 2, figs. 11–14 (?); Harmer 1926: 412, pl. 28, figs. 1–4 (?); Calvet 1927: 4 (?); Gautier 1962: 97; Prenant and Bobin 1966: 555, fig. 191; Zabala 1986: 333, fig. 96; Zabala and Maluquer 1988: 101, fig. 176; Hayward and McKinney 2002: 24, fig. 10 A–B

### Material examined

*Holotype*: MHNUSC 10104, Brijuni Island, Croatia, Adriatic Sea, 44° 54′ 04″ N, 13° 46′ 07″ E, 20-m depth, 03/06/2017, on calcified red seaweeds.

*Paratypes*: MHNUSC 10105, Zlatne Stijene, Pula, Croatia, Adriatic Sea, 45° 50′ 11″ N, 13° 49′ 35″ E, 24-m depth, 03/06/2017, on calcified red seaweeds.

*Other material*: **Balearic Islands** (**Mediterranean**)—MNCN 25.03/557, 25.03/736, I. Conejera, Ibiza, Loc. 259B, FAUNA III, 35 m; MNCN 25.03/1132, I. Tagomaro, Ibiza, Loc. 236B4, FAUNA III, 17.3-m depth; MNCN 25.03/1658, Isla Bleda Mayor, Ibiza, Loc. 258B3, FAUNA III, 6–45-m depth; MNCN 25.03/675, Pta. Jova, Mallorca, Loc. 179 B1, FAUNA III, 13.3-m depth; MNCN 25.03/712, I. de Toro, Mallorca, Loc. 230B14, FAUNA III, 30-m depth; MNCN 25.03/1072, Pta. de La Guardia, Mallorca, Loc. 222 A, FAUNA III, 92–97-m depth; MNCN 25.03/1095, Isla de Dragonera, Mallorca, Loc. 177B2, FAUNA III, 31.3-m depth. **Iberian Peninsula** (**Mediterranean**)—MNCN 25.03/3107, Islas Columbretes, Castellón, Loc. 293A, FAUNA IV, 78–81-m depth; MNCN 25.03/3158, Islas Columbretes, Castellón, Loc. 283A, FAUNA IV, 80–85-m depth. **France** (**Mediterranean**)—MNHN 1173, Banyuls-sur-Mer, 06/vi/1962; NHMUK 1975.4.20.5, Cassis, Marseille, Jean-Georges Harmelin Collection, 30-m depth, 27/viii/1974; MZUSP 1312, Montremian, Port-Cros Island, collector Jean-Georges Harmelin, 27-m depth, 05/v/2016. **Italy** (**Ligurian Sea**)—NHMUK 1968.1.16.2, Santa Margherita, Riviera Levante; (**Tyrrhenian Sea**) NHMUK 1879.4.25.45, Naples, Waters Collection. **Adriatic Sea**—NHMUK 1911.10.1.304; NHMUK 1912.12.21.923, Norman Collection. **Greece** (**Aegean Sea**)—NHMUK 1975.1.12.118/123/124/128/130, Cape Mastika, Chios, Expedition to Chios, University College Swansea, viii/1967; NHMUK 1975.1.12.122/125/129, Venetica, Chios, Expedition to Chios, University College Swansea, viii/1967; NHMUK 1975.1.12.122, Dhiaporia, Chios, Expedition to Chios, University College Swansea, viii/1967.

*Etymology*: Alluding to the presence of this species in Mediterranean waters.

### Description

Colonies encrusting, forming a sheet with network aspect, supported above the substrate, and fixed to it, by rootlets with holdfast tip. Autozooids disjunct, each zooid with six tubular connections, one distal, two distolateral, two proximolateral, and one proximal, a septum, visible by transparency, in the middle of the tubular connections, with numerous small rounded pores forming a circle. Autozooids large, elongate oval, boat-shaped, with frontal wall completely membranous. No true oral spines present, two or four small distal projections occasionally present, which may move to the distal corners in the presence of an ooecium. Distal border of autozooids rounded, with edge of operculum fitting to the distal rim. One or two monomorphic avicularia, pedunculate, with bird’s head form and very mobile, attached by a short tube near each distolateral tubular connection and directed frontally, elongate cystid, almost funnel-shaped, with dorsal wall rounded in profile, rostrum downcurved distally, hooked, without denticulate border. Ooecium very reduced, detected only by the presence of a small distal cap with small uncalcified distolateral areas. 24–28 tentacles.

### Remarks

Samples here ascribed to *B. mediterranea* sp. nov. come from different Mediterranean areas: the Balearic Islands, Spanish Levante (Castellón), Southern France (Banyuls-sur-Mer, Marseille, and Port-Cros Island), Southern Italy (Naples), the Northern Adriatic and the Eastern Mediterranean (Chios). All this material differs from *B. magellanica* and *B. serrata* sp. nov. in the absence of oral spines as well as bigger zooids and avicularia, which lack a denticulate rostrum. Specimens assigned to *B. magellanica* by Hayward and McKinney (2002) from the Adriatic and by Prenant and Bobin (1966) and Zabala (1986) from the western Mediterranean definitely correspond to *B. mediterranea* sp. nov. Prenant and Bobin (1966) and Zabala (1986) indicated the presence of distal spines in the autozooids, though vestigial. The figure by Prenant and Bobin (1966, fig. 191) actually shows only two distal shallow folds, while the key by Zabala and Maluquer (1988) indicates that *B. magellanica* lacks spines or that they are vestigial. On the other hand, Waters (1879) reported that “Just above the aperture are from two to four small projections on the zooecium” in material from Naples, but he did not refer to real spines. The revision of Waters’ original specimens in the NHMUK proves the lack of true spines in this material, which is also referred here to *B. mediterranea* sp. nov.

The identity of other nominal records of *B. magellanica* from the Mediterranean could not be confirmed and should be revised, including those from the Adriatic (Heller 1867; Novosel et al. 2004; Cocito et al. 2006), Italy (Waters 1897; Harmer 1926; Rosso et al. 2010; Rosso and Di Martino 2016), Balearic Islands (Calvet 1927; Zabala 1993); Banyuls-sur-Mer (Calvet 1927), Islas Columbretes (d’Hondt 1979; Saguar and Boronat 1987), Lebanon (Harmelin et al. 2016), and Greece (Gerovasileiou and Rosso 2016). Nonetheless, most of those records likely correspond to *B. mediterranea* sp. nov.

## Discussion

Besides the variability described in our material and included in the above results, certain other variations in the material examined as well as in the descriptions from the literature were observed, although these probably lack taxonomic meaning. For instance, one or two avicularia may be present in different zooids in our material (as also described or figured in the literature (e.g., Prenant and Bobin 1966; Hayward and McKinney 2002)), but a real pattern was not observed. Also, the length of the tubular connections between autozooids is very variable, with proportionally the highest standard deviation of all the measurements taken (Table 1). This variation probably depends on the substrate because colony morphology varies according to substrate heterogeneity. In the western Mediterranean localities studied here, all specimens were found on calcareous algae, but in the Adriatic, specimens were found on bryozoans (*Schizoretepora serratimargo* (Hincks, 1886)) as well as on calcareous algae. In the NW Mediterreanean, *B. magellanica* s.l. is particularly common in coralligenous grounds where it is epibiont of many taxa, such as coralline algae and sponges (Harmelin 2017). In northern Spain, colonies of *B. serrata* sp. nov. grew on the alga *G. repens* and on the bryozoan *S. cervicornis* (no substrate data are available for specimens deposited at the NHMUK). This variability in the length of the tubular connections was also observed in other bryozoan species, such as genus *Mollia* Lamouroux, 1816 (Berning 2006; Lamouroux 1816; Souto et al. 2010a), with a similar colonial development, i.e., fixed to the substrate by rootlets that avoid contact of the basal wall with the substrate and settlement on similar substrates, mainly coralline algae.

**Fig. 7:**
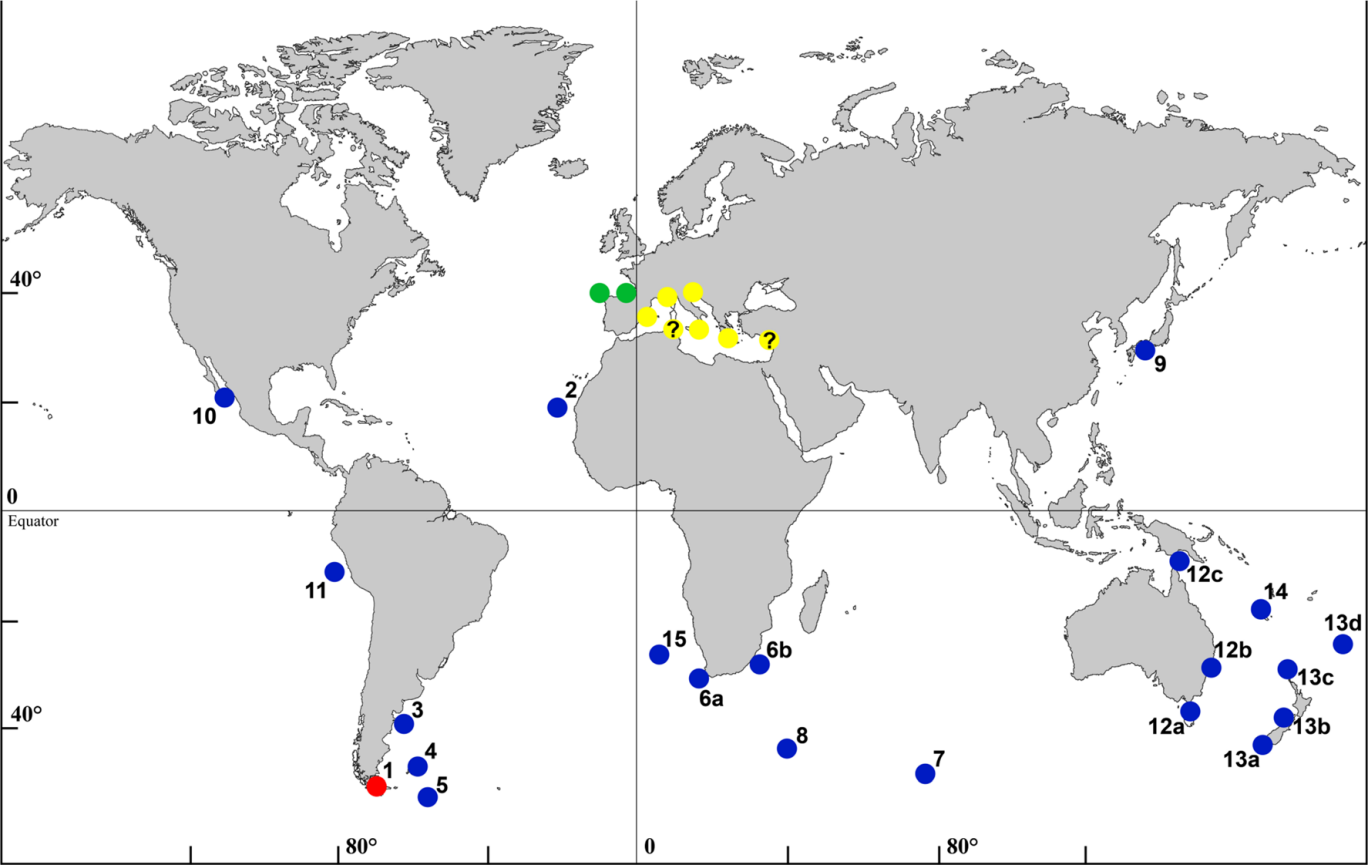
Distribution of *Beania magellanica* s.l. Red circle, type locality of *B. magellanica*; blue circles, summary of localities with *B. magellanica* s.l. records in areas not studied in the present paper; green circles, localities of *B. serrata* sp. nov.; yellow circles, localities of *B. mediterranea* sp. nov., with question mark (?), localities with record as *B. magellanica*, and that could probably be *B. mediterranea*, but whose identity should be checked. References from areas or samples not included in the present paper: (1) Magellan Strait, Chile (Busk 1852; Waters 1904; Hastings 1943; Moyano 1999, 2009); (2) Cape Verde (Jullien 1888; Calvet 1907); (3) Argentina (Jullien 1888; Hastings 1943; Moyano 2005; López-Gappa 1989; Bastida et al. 1992, Liuzzi and López-Gappa 2011); (4) Falkland Islands (Ortmann 1890; Hastings 1943; Moyano 2005); (5) Burdwood Bank (Hastings 1943); (6) South African coasts, a (Jullien 1888; O’Donoghue and de Watteville 1944; O’Donoghue and Day 1957; Florence et al. 2007) and b (Hayward 1980; Hayward and Cook 1983); (7) Kerguelen (Busk 1879; Kluge 1914); (8) Marion and Prince Edward Islands (Hastings 1943; Hayward 1995); (9) Pacific coast of Japan (Jullien 1888; Ortmann 1890; Yanagi and Okada 1918; Harmer 1926; Silén 1941); (10) Mexico (Soule 1959); (11) Peru (Osburn 1950, 1953); (12) a–c, Australia (Hincks 1885; MacGillivray 1887; Harmer 1926; Vail and Wass 1981; Bock 1982; Winston 1986; Moran and Grant 1989, 1991, 1993; Connell 2001; Glasby et al. 2007; Piola and Johnston 2008); (13) a–d, New Zealand (Busk 1852; Hutton 1891; Hamilton 1898; Waters 1906; Livingstone 1927; Gordon 1970, 1984, 1986; Bradstock and Gordon 1983; Rowden et al. 2004; Gordon et al. 2009); (14) New Caledonia (Gordon 2007); (15) Vema seamount, JS, unpublished data

As noted in the introduction, *B. magellanica* has so far been considered a widespread species, reported from all around the world (Fig. 7). Nonetheless, all these records should be revised according to the new data presented here. The observed biometric differences and the morphological differences in some characters (presence, absence, and number of oral spines; number of spines in ovicelled zooids; size and morphology of avicularium, presence of a serrated or smooth rostrum) lead us to describe two new species from the Atlantic-Mediterranean region, and also to confirm that *B. magellanica* actually represents a species complex.

Furthermore, *B. magellanica* s.s. is a species that could have been transported by anthropogenic means in the past. Moyano (2009) reported that *B. magellanica* was the most common *Beania* species in the Chilean Fjordland and Patagonia, and considered it to have been introduced from Patagonia by vessels. Specimens identified as *B. magellanica* s.l. were found on artificial experimental substrates on the coast of Sydney, Australia (Vail and Wass 1981; Moran and Grant 1989, 1991, 1993).

The first find of a new species, here described as *B. serrata* sp. nov., belonging to *B. magellanica* s.l. in the NE Atlantic, may be misleading. Some small species sampled from cryptic habitats have been considered native in the type localities, as in the case of some pseudo-indigenous species (Carlton 2009; Rocha et al. 2013; Marchini et al. 2015; Miranda et al. 2018). At the same time, the lack of data from the sampled area may have led to the misassignment of the exotic status in various marine species (Zenetos et al. 2017), including bryozoans (Rocha et al. 2013; Miranda et al. 2018).

Thus, the absence of previous records of *B. magellanica* s.l. from the European Atlantic coast suggests that *B. serrata* sp. nov. was recently introduced here by anthropogenic activities. The Ría de Ferrol is home to major shipyards and a naval industry, and Point Etxandarri is close to the harbor of Bilbao. This causes both areas to have high anthropogenic pressure through commercial and leisure vessel traffic. The fauna of both areas is well-known, in particular that of the Ría de Ferrol, which has been systematically, at least yearly, surveyed since about 25 years ago. Until now, *B. magellanica* s.l. was never recorded there, so most probably the species was introduced during recent years. This is not the first time that a new bryozoan immigrant has been detected in that area. During the 1990s, a widespread species, *Watersipora subatra* (Ortmann, 1890), was detected for the first time in the nearby coast of Lugo (César-Aldariz et al. 1997 as *W. subovoidea*), and only a few years ago, it began to spread along the whole north Iberian coast (JS, unpublished data). Also, during the 1990s, *Tricellaria inopinata* d’Hondt and Occhipinti Ambrogi, 1985 was first detected at the coast of Lugo, and it has now colonized the whole Galician coast as well as the Bay of Biscay (De Blauwe and Faasse 2001; Fernández-Pulpeiro et al. 2002; Cook et al. 2013). Another well-known invasive species, *Amathia verticillata* (Delle Chiaje, 1822), was detected in Galicia and Santander for the first time a few years ago (Souto et al. 2010b; Reverter-Gil et al. 2016). Finally, several well-known invasive species of the genera *Bugula* and *Bugulina* are present along the whole north Iberian coast (JS and ORG, unpublished data).

## Conclusions

Although *B. magellanica* is considered a widespread species, which has probably been transported by human activities, there are some misidentified specimens around the world and it apparently represents a large species complex of closely related species. Here, we describe two new species closely resembling *B. magellanica*: *B. serrata* sp. nov. from the Atlantic coast of northern Spain, and *B. mediterranea* sp. nov. from the Mediterranean coast. The latter species was previously identified as *B. magellanica*, whereas the *B. serrata* sp. nov. was probably recently introduced into the area. The presence of unknown species described outside of their original distribution is not exceptional among bryozoans because several other species were described directly as non-indigenous (e.g., d’Hondt and Occhipinti-Ambrogi 1985; Souto et al. 2018; Miranda et al. 2018). Thus, we consider it as to be cryptogenic. Revision of this entire species complex will no doubt yield more new species and perhaps also help clarify the origin of *B. serrata* sp. nov.
